# Histopathological Analysis of Nodal Disease After Chemoradiation Reveals Viable Tumor Cells as the most Important Prognostic Factor in Head and Neck Squamous Cell Carcinoma

**DOI:** 10.1007/s12105-023-01557-7

**Published:** 2023-05-17

**Authors:** Aline Golliez, Grégoire B. Morand, Martina A. Broglie, Panagiotis Balermpas, Niels J. Rupp

**Affiliations:** 1 Department of Pathology and Molecular Pathology, University Hospital Zurich, Zurich, Switzerland; 2 Department of Otorhinolaryngology - Head and Neck Surgery, University Hospital Zurich, Zurich, Switzerland; 3 Department of Otolaryngology - Head and Neck Surgery, Sir Mortimer B. Davis – Jewish General Hospital, McGill University, Montreal, QC Canada; 4 Faculty of Medicine, University of Zurich, Zurich, Switzerland; 5 Department of Otorhinolaryngology, Head and Neck Surgery, Lucerne Cantonal Hospital, Lucerne, Switzerland; 6 Department of Radiation Oncology, University Hospital Zurich, Zurich, Switzerland

**Keywords:** Neck Dissection, Squamous Cell Carcinoma of Head and Neck, Prognosis, Lymph Nodes, Keratins

## Abstract

**Background:**

In head and neck squamous cell carcinoma (HNSCC), salvage neck dissection (ND) is required after primary chemoradiation in case of residual nodal disease. Upon histopathological examination, viability of tumor cells is assessed but little is known about other prognostic histopathological features. In particular, the presence of swirled keratin debris and its prognostic value is controversial. The aim of this study is to examine histopathological parameters in ND specimens and correlate them with patient outcome to determine the relevant parameters for histopathological reporting.

**Materials and Methods:**

Salvage ND specimen from a cohort of n = 75 HNSCC (oropharynx, larynx, hypopharynx) patients with prior (chemo) radiation were evaluated on H&E stains for the following parameters: viable tumor cells, necrosis, swirled keratin debris, foamy histiocytes, bleeding residues, fibrosis, elastosis, pyknotic cells, calcification, cholesterol crystals, multinucleated giant cells, perineural, and vascular invasion. Histological features were correlated with survival outcomes.

**Results:**

Only the presence / amount (area) of viable tumor cells correlated with a worse clinical outcome (local and regional recurrence-free survival, (LRRFS), distant metastasis-free survival, disease-specific survival, and overall survival, p < 0.05) in both the univariable and multivariable analyses.

**Conclusion:**

We could confirm the presence of viable tumor cells as a relevant negative prognostic factor after (chemo) radiation. The amount (area) of viable tumor cells further substratified patients with worse LRRFS. None of the other parameters correlated with a distinctive worse outcome. Importantly, the presence of (swirled) keratin debris alone should not be considered viable tumor cells (ypN0).

**Supplementary Information:**

The online version contains supplementary material available at 10.1007/s12105-023-01557-7.

## Introduction

For locally advanced head and neck squamous cell carcinomas (HNSCC), primary chemoradiation is the standard of care first-line therapy as an organ preservation strategy [1]. A surgical approach with a neck dissection (ND) may then be required for the patients with residual disease or nodal disease persistence, especially if metabolically active on FDG-PET-CT scan [2], [3]. Staging according to the UICC Guidelines encompasses cervical lymph node status [4]; however, the prognostic value following chemoradiation and neck dissections, is still investigated. Patients with histologically confirmed positive lymph nodes including viable tumor cells after chemoradiation have been demonstrated to show a worse outcome [5]. This makes viable tumor cells in lymph nodes examined after salvage neck dissection an important prognostic factor for patient outcome.

However, there is a lack of a widely accepted and clearly defined histological consensus for definition of viable and non-viable tumor cells in lymph nodes [5], [6]. In the case of nucleated keratin debris, it must be assumed that it is a derivative of tumor cells; however, only very limited data on the significance for the patient is available [7].

This leaves the classification of histological features evaluated in a pretreated lymph node to the individual pathologist.

This study analyzed a variety of histopathological features of head and neck squamous cell carcinomas in cervical lymph nodes obtained from salvage neck dissection performed after primary (chemo) radiation. We delineated viable tumor cells histologically from other features indicating a treatment effect, including swirled keratin debris and pyknotic cells. Each histopathological feature was correlated with patient outcome to determine whether features other than viable tumor cells should be reported by pathologists.

## Materials and Methods

### Study Population

We used the study cohort previously described by Rüegg et al., which consists of patients with oropharyngeal, laryngeal, and hypopharyngeal squamous cell carcinoma that underwent primary chemoradiation followed by a salvage neck dissection [8]. We did not include oral cavity squamous cell carcinoma as these tumors most often undergo primary surgery followed by radiation as required. A retrospective assessment was performed after an approval of the Ethics Review *Board Kantonale Ethikkomission Zürich* (*protocol number 2016 − 01799*). All patients were treated at the Department of Otorhinolaryngology–Head and Neck Surgery, University Hospital Zurich. A salvage neck dissection was performed on all patients with persisting nodal disease after primary chemoradiation. This was defined by the presence of FDG-active lymph nodes or lymph nodes larger than 1 cm in short-axis measurement in a PET scan performed three months after chemoradiation [3]. Excluded from the study were patients with induction chemotherapy, patients who did not complete a course of locoregional radiotherapy with a dose of at least 60 Gy, and patients with a primary surgical approach. The staging was performed according to the *Union Internationale Contre le Cancer* (UICC) and TNM staging for head and neck cancer, seventh edition, 2010 [4]. All patients were discussed at the local multidisciplinary tumor board after obtaining a full medical history, physical examination, triple endoscopy with biopsy (pharyngo-laryngoscopy, trachea-bronchoscopy, and esophagoscopy), and imaging with FDG-PET/CT or FDG-PET/MR. Data collected included patient age, gender, tumor subsite, and risk factors, including smoking, drinking habits, and HPV status. A diffuse, “block-type” p16INK4A overexpression in tumor tissue by immunohistochemistry served as a surrogate marker for HPV-driven carcinogenesis of oropharyngeal squamous cell carcinoma [9]. Further, the local and regional radiation dose, type and number of cycles of concomitant chemotherapy, time to follow-up FDG-PET, pathological tumor stage, number of nodes dissected, and follow-up time were assessed.

The outcome measures obtained were local and regional recurrence-free survival (LRRFS), distant metastasis-free survival (DMFS), disease-specific survival (DSS), and overall survival (OS).

Ultimately, a total of seventy-eight cases (*n* = 78) were included in the study.

### Neck Dissection and Selection of Representative Lymph Node

A selective salvage neck dissection was performed on all of the patients at the Department of Otorhinolaryngology, Head and Neck Surgery, University Hospital Zurich, representing the standard of care for patients with persisting nodal disease after the primary chemoradiation [10]. We included only the primary neck dissection for the individual patient, excluding any other neck dissections, which might be performed during the further course. The excised lymph nodes were assessed in the Department of Pathology, University Hospital Zurich, and the staging was performed according to the UICC, TNM staging for head and neck cancer, seventh edition, 2010 [4]. For our study, we examined all diagnostic hematoxylin and eosin (H&E) slides for every patient. The H&E slide showing the lymph node with the largest lesional tissue was determined. This process was carried out in the form of a double reader assessment. If there were no viable tumor cells, the lymph node with the most severe reaction was selected. If viable tumor cells were detectable, the lymph node with the largest extension was selected. In summary, we determined the worst case as our representative slide for that patient.

### Assessment of the Lymph Nodes and Histopathological Criteria

The selected slides were scanned using the Hamamatsu NanoZoomer Scanner and assessed using the viewing software NDP.view2 version 2.8. by Hamamatsu.

For every selected slide, a number of histological features were assessed and in the second step reviewed, making this a double reader assessment.

**Fig. 1:**
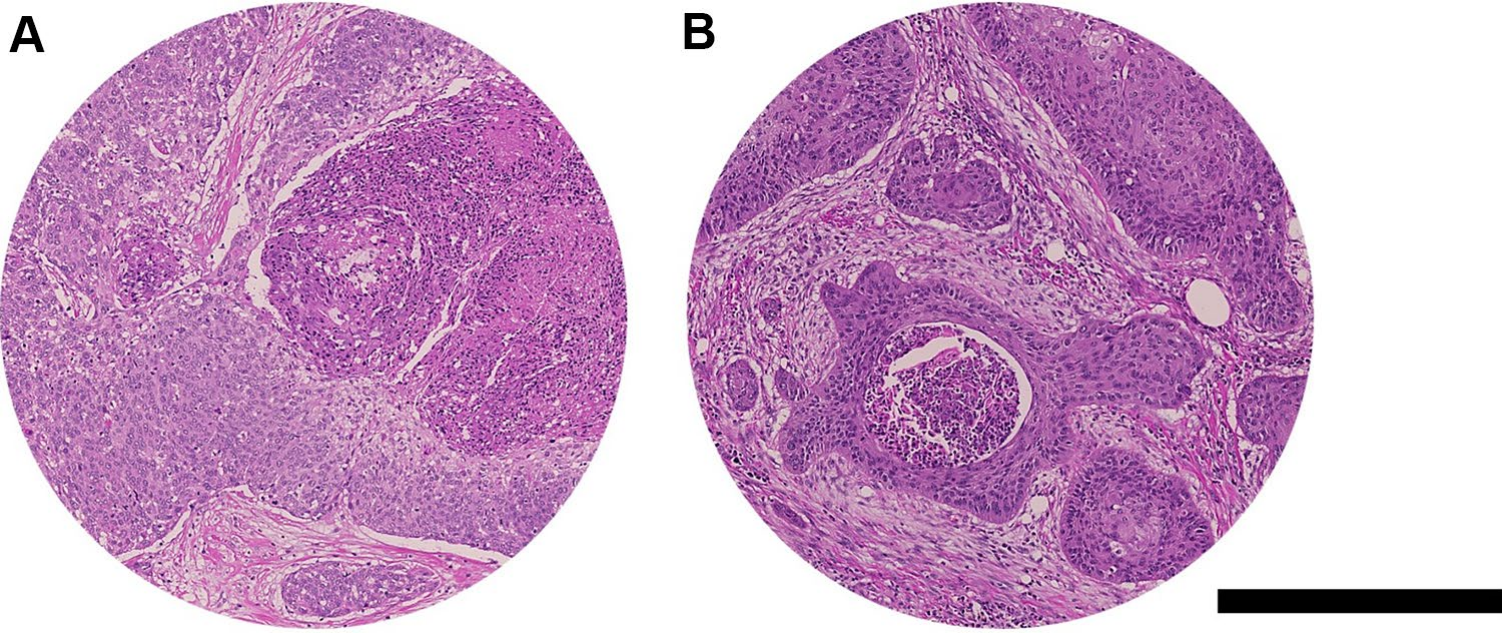
Histological appearance of viable tumor cells in lymph nodes from salvage neck dissections after primary chemoradiation (**A**, **B**, hematoxylin and eosin (H&E)). Highly atypical squamous cells are depicted with comedo-like necrosis and retained nuclei. Scale bar: 250 μm

**Fig. 2:**
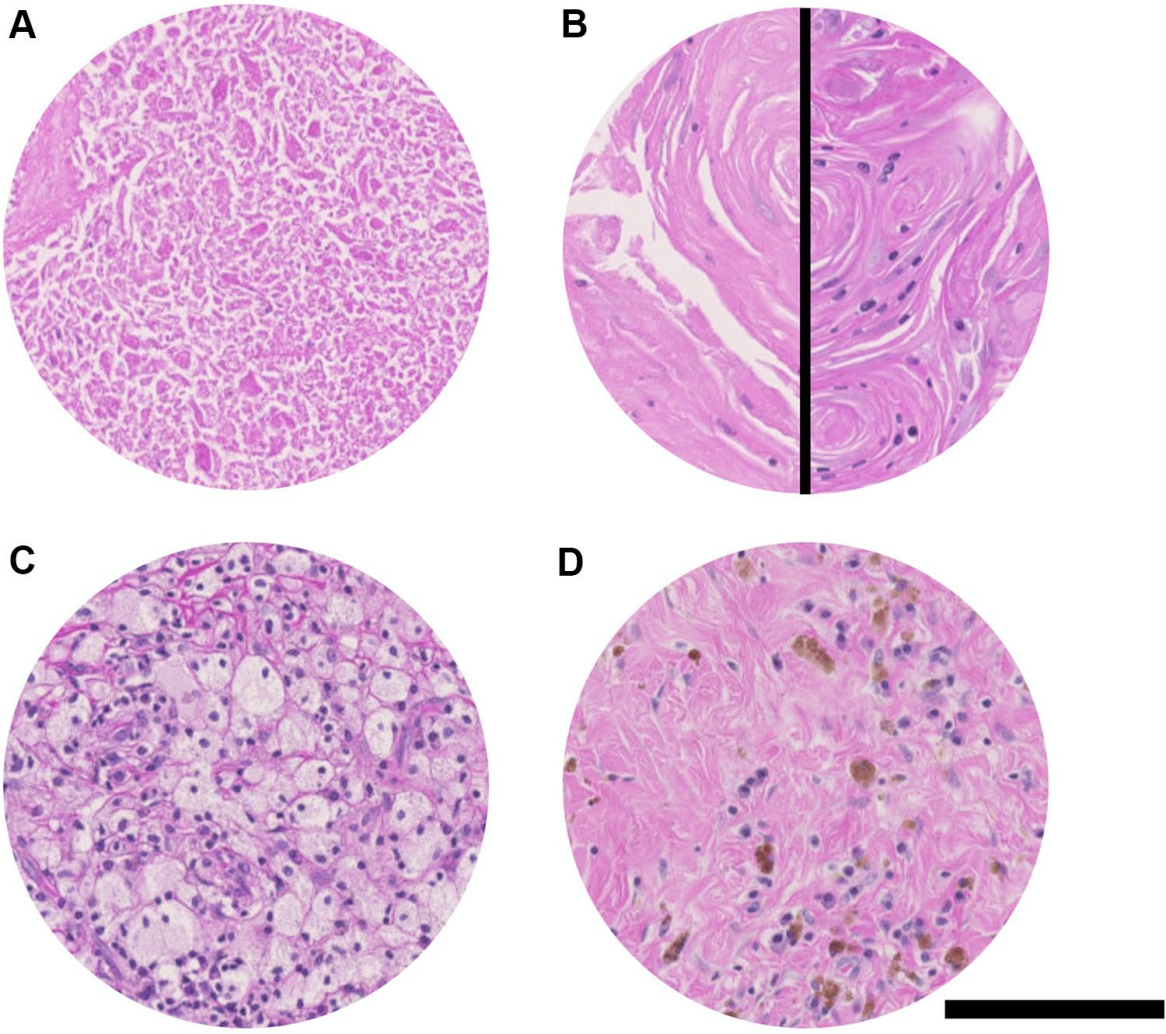
In the absence of viable tumor cells, there are various histopathological features indicating some kind of treatment effect, such as necrosis (**A**) or swirled keratin debris (**B**). We subdivided the swirled keratin debris (SKD) in our evaluation in < 50% nucleated SKD (B, left side) and ≥ 50% nucleated SKD (B, right side). Furthermore, there were foamy histiocytes (**C**) and bleeding residues (**D**) present in some of the histologically evaluated lymph nodes. Hematoxylin and Eosin (H&E) staining. Scale bar: 250 μm

Viable tumor cells were defined as highly atypical squamous cells with typical eosinophilic cytoplasm and irregular nuclei as shown in Fig. 1A and B; this definition is in concordance with a previous publication from another group [7]. With viable tumor cells present, we also assessed the expansion in mm^2^ on the selected slide and determined if there was any extranodal expansion or not. Histopathological features indicating a treatment effect encompassed the presence of necrosis and swirled keratin debris (Fig. 2A–B). For those two characteristics, apart from a qualitative assessment, we measured the maximum diameter and the expanse as a quantitative assessment (mm/mm^2^). These parameters were divided into ordinal categories for semi-quantitative statistical analysis. The swirled keratin debris was qualitatively further subdivided in ≥ 50% and < 50% nucleated swirled keratin debris. The swirled keratin debris was defined histologically as lamellated keratin with an eosinophilic aspect and hematoxylin-positive nuclei absent or present as shown in Fig. 2B; this definition was similar to the previous publication from another group [7].

Other histopathological features indicating a treatment effect such as histiocytes and bleeding residues were assessed qualitatively.

**Fig. 3:**
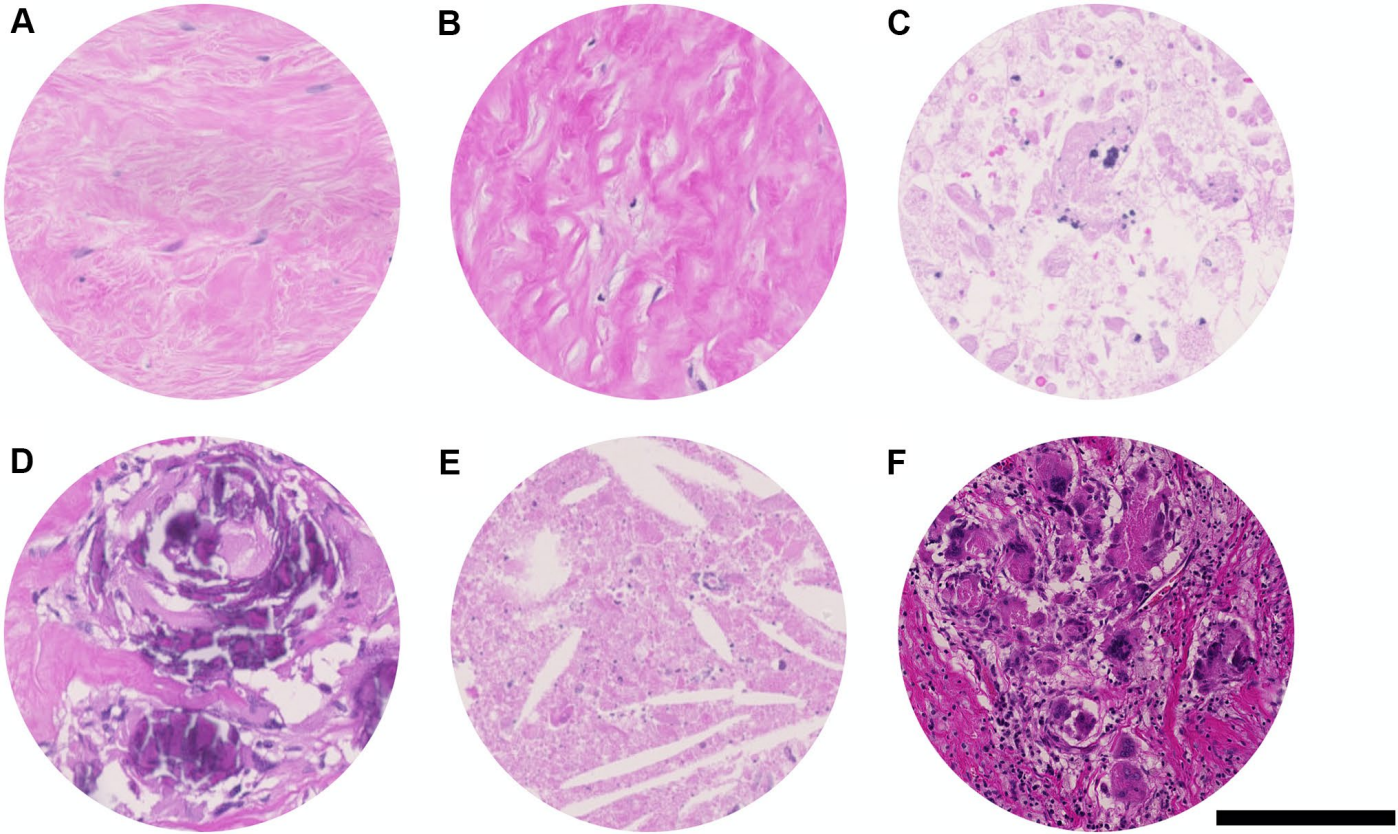
Further evaluated histopathological features included fibrosis (**A**), elastosis (**B**), and pyknotic cells found within necrosis (**C**). Calcification (**D**), cholesterol crystals (**E**), and multinucleated giant cells (**F**) were also evaluated as reaction patterns. Hematoxylin and Eosin (H&E) staining. Scale bar: 250 μm

Further evaluated histopathological features (Fig. 3) included fibrosis and elastosis. In addition, pyknotic cells found within necrosis, calcifications, cholesterol crystals, and multinucleated giant cells as histopathological features representing a reaction pattern were assessed. Furthermore, also perineural invasion and vascular invasion of viable tumor cells were assessed; however, due to the low case numbers (three cases with vascular invasion and one case with perineural invasion), the data were not included in the statistical analysis.

### Statistical Analysis

For continuous variables, distribution was evaluated for normality according to Gauss theorem. For normally distributed variables, mean and standard deviations are given. For non-normally distributed variables, median and interquartile range (IQR) are given. Survival curves were built according to Kaplan–Meier, and the log-rank test was used to compare factors. For multivariable analysis, a Cox regression model was built integrating all relevant factors. A *P*-value lower than 0.05 was considered to indicate statistical significance. Statistical analyses will be performed using SPSS® 27.0.0.1 software (IBM®, Armonk, NY, USA).

## Results

### Patient and Tumor Characteristics

The details of the study cohort have been previously described by Rüegg et al. [8]. Briefly, the cohort consisted of n = 78 patients with laryngeal, hypopharyngeal, or oropharyngeal cancer undergoing salvage neck dissection after primary chemoradiation. There were *n* = 68 (87%) men and *n* = 10 (13%) women. The tumor localization was predominantly in the oropharynx *n* = 56 (73%), followed by the hypopharynx *n* = 14 (18%) and the larynx *n* = 7 (9%). More than half of the patients were staged as cT3–cT4 Tumors (53%) in comparison to cT1–cT2 (47%). The clinically assessed lymph node status was positive in *n* = 72 patients (92%) with *n* = 55 (71%) of them having cN1–cN2b classification and only *n* = 17 (22%) with cN2c–cN3. The median of the follow-up time for all patients was 37 months.

Of the 56 patients with oropharynx as the primary tumor site, 56.4% patients with available information were positive for p16 staining, meanwhile 43.6% were p16 negative.

### Descriptives of Histopathological Parameters

The resected lymph nodes after the salvage neck dissection were assessed first determining the representative slide for each patient as previously described and then evaluated for the various histopathological criteria (Table 1).

**Table 1:** Description of histopathological parameters assessed on the representative slide for each patient

Pathological criteria	Study cohort *N* = 75	Mean	Std. deviation
Viable tumor cells			
No (%)	45 (60)		
Yes (%)	30 (40)		
Area mm^2^		16.8	32.3
Extranodal extension			
No (%)	62 (82,7)		
Yes (%)	13 (17.3)		
Necrosis			
No (%)	24 (32)		
Yes (%)	52 (68)		
Area mm^2^		16.1	33
Max. Diameter mm		2.8	4.4
Swirled keratin debris			
No (%)	57 (76)		
< 50% (%)	11 (14.7)		
≥ 50% (%)	7 (9.3)		
Area mm^2^		16.6	49.2
Max. diameter mm		2.2	5.1
Foamy Histiocytes			
No (%)	54 (72)		
Yes (%)	21 (28)		
Bleeding residues			
No (%)	60 (80)		
Yes (%)	15 (20)		
Fibrosis			
No (%)	16 (21.3)		
Yes (%)	59 (78.7)		
Area mm^2^		92.8	98.4
Max. diameter mm		12.1	12.1
Elastosis			
No (%)	54 (72)		
Yes (%)	21 (28)		
Pyknotic cells			
No (%)	33 (44)		
Yes (%)	42 (56)		
Calcification			
No (%)	48 (64)		
Yes (%)	27 (36)		
Cholesterol crystals			
No (%)	54 (72)		
Yes (%)	21 (28)		
Multinucleated giant cells			
No (%)	21 (68)		
Yes (%)	24 (32)		
Perineural invasion			
No (%)	74 (99)		
Yes (%)	1 (1)		
Vascular invasion			
No (%)	72 (96)		
Yes (%)	3 (4)		

Tissue blocks/slides from *n* = 75 patients were available, meaning 3 out of the 78 Patients (3.8%) from the study cohort from Rüegg et al. [8] were excluded from our study. Of the patients evaluated, we found viable tumor cells in *n* = 30 cases (40%) with a mean area measured of 16.8 mm^2^ (± 32.3) in comparison to *n* = 45 patients (60%) with no viable tumor cells present. There was an extranodal extension in *n* = 13 patients (17.3%), so 43.3% of cases with viable tumor cells also showed an extranodal extension. Of the histopathological features indicating some kind of treatment effect, necrosis was shown in *n* = 52 patients (68%) with a mean area of 16.1 mm^2^ (± 33) and mean maximal diameter of 2.8 mm (± 4.4). Swirled keratin debris was present in *n* = 18 cases (24%), of which *n* = 11 (14.7%) were < 50% and *n* = 7 (9.3%) were ≥ 50% nucleated. Quantitatively, the swirled keratin debris showed a mean area of 16.6 mm^2^ (± 49.2) and mean maximal diameter of 2.2 mm (± 5.1). Further, foamy histiocytes were present in *n* = 21 (28%) and bleeding residues in *n* = 15 (20%) cases. Fibrosis was shown in *n* = 59 patients (78.7%) with a mean area of 92,8 mm^2^ (± 98.4) and a mean maximal diameter of 12.1 mm (± 12.1). Elastosis was present in *n* = 21 (28%), pyknotic cells in *n* = 42 (56%), calcifications in *n* = 27 (36%), cholesterol crystals in *n* = 21 (28%), and multinucleated giant cells in *n* = 24 (32%) cases. Perineural invasion was only detected in *n* = 1 (1,3%) and vascular invasion in *n* = 3 patients (4%). Due to the low numbers, we did not include these two parameters in our further statistical analysis.

### Univariable Survival Analysis

Kaplan–Meier curves were created for the assessed histopathological parameters for each of the four outcome measures and an univariable log-rank test was performed (Supplementary Table 1).

**Fig. 4:**
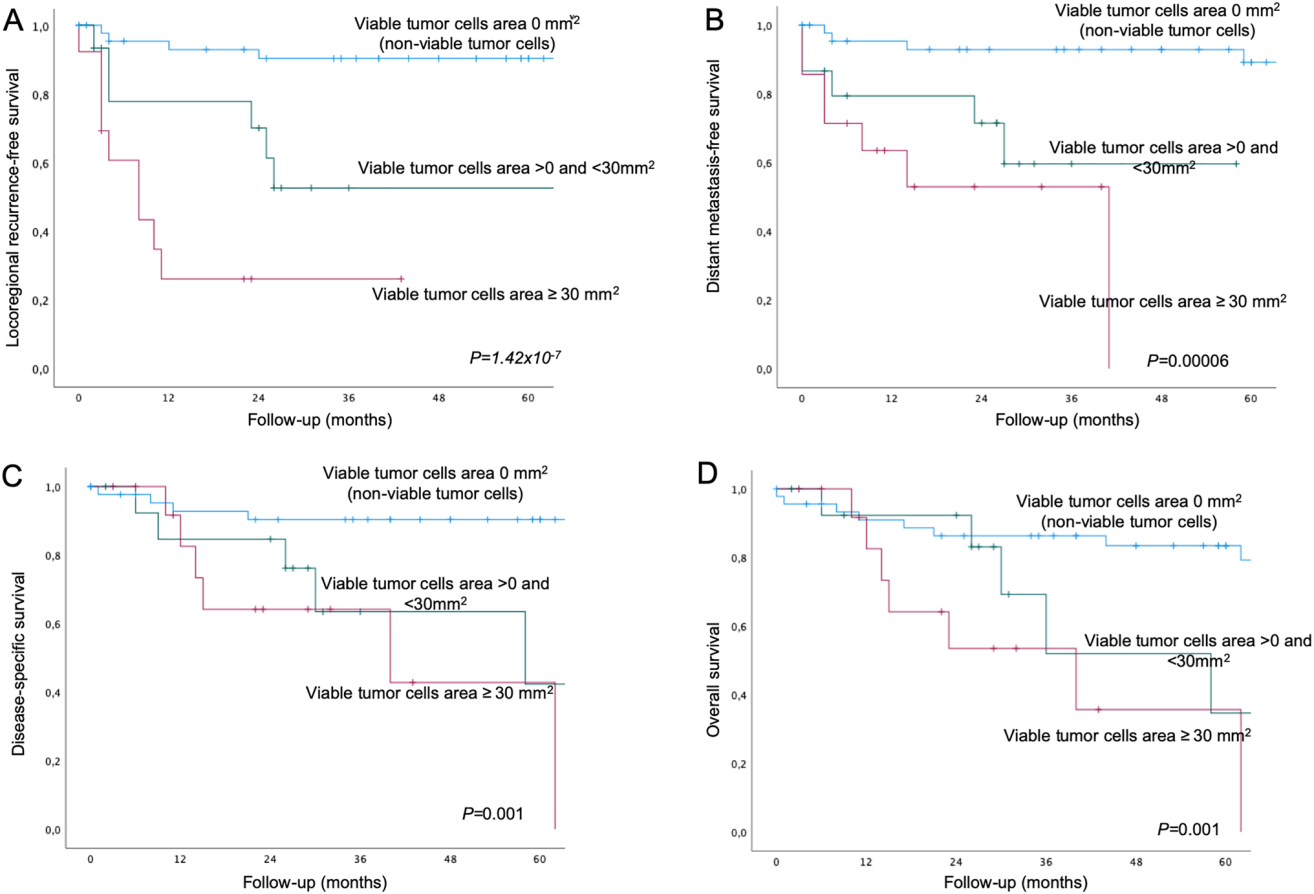
Survival curves according to viable tumor cells area of salvage neck dissection specimen, shown for (**A**) locoregional recurrence, (**B**) distant metastasis-free survival, (**C**) disease-specific survival, and (**D**) overall survival. Respective *P*-values are indicated on each quadrant (Log-rank test). NB: In panel A, the relative locoregional recurrence-free survival of patients with viable tumor cells on an area < 30 mm^2^ and viable tumor cells on an area ≥ 30 mm^2^ was also statistically significant (log-rank, *P* = 0.039) in direct comparison (excluding non-viable cells)

The locoregional recurrence-free survival, distant metastasis-free survival, disease-specific survival, and overall survival were significantly worse in patients with presence of viable tumor cells (log-rank test, *P* = 3.53 × 10^− 7^, *P* = 0.000005, *P* = 0.00001, and *P* = 0.0001, respectively) (Supplementary Fig. 1). Stratification of the area of viable tumor cells into no viable cells (area = 0 mm^2^), viable tumor cells with an area > 0 but < 30 mm^2^, and viable tumor cells with an area ≥ 30 mm^2^ (Fig. 4) revealed a highly significant separation of the three survival curves for locoregional recurrence-free survival (*P* = 1.42 × 10^− 7^). Comparison between viable tumors cells with an area > 0 but < 30 mm^2^ and viable tumor cells with area ≥ 30 mm^2^ also showed to be statistically significant (Log rank, P = 0.039).

The respective *P*-values of the other parameters in univariable analysis are shown in Supplementary Table 1. Due to low case numbers of perineural and vascular invasion no statistical analysis was possible and therefore performed.

We performed a subgroup analysis of patients with oropharyngeal squamous cell carcinoma only. Patients with p16-positive oropharynx tumors were less likely to have viable tumors cells in salvage neck dissection samples (OR 0.09, 95%CI 0.02–0.44, *P* = 0.003). Conversely, p16-positive tumors had significantly better locoregional recurrence-free survival, disease-specific survival, and overall survival (*P* = 0.038, P = 0.007, and *P* = 0.021, respectively). 

### Multivariable Survival Analysis

For a more in-depth analysis of the potential relevant prognostic histopathological features, a multivariable Cox regression survival analysis was performed to minimize the effect of potential confounders and to reveal potential independent prognostic factors. For the multivariable analysis, we included all factors that were clinically and/or in univariable analysis statistically relevant, i.e., “viable tumor cells,” “extranodal extension,” “swirled keratin debris,” “foamy histiocytes,” and “fibrosis.”

In multivariable Cox regression analysis, presence of viable tumor cells was the only independent prognostic factor for locoregional recurrence-free survival, distant metastasis-free survival, disease-specific survival, and overall survival (*P* = 0.01, *P* = 0.02, *P* = 0.00009, and *P* = 0.0002, respectively). All other included cofactors were not significant in all 4 main outcome survival analyses (*P* > 0.05).

## Discussion

Salvage neck dissection is the standard of care in patients with residual nodal disease after the established first-line therapy of primary chemoradiation [2]. Pathologists conduct a histopathological examination of the resected lymph nodes stating the presence or absence of viable tumor cells, which then determines the cervical lymph node status according to the UICC Guidelines (ypN0 vs. ypN+) [4]. However, there is no consensus on the prognostic value of other histopathologic features, which are also frequently assessed in the pathology report. Furthermore, there is no clearly defined cut-off from viable to non-viable tumor cells, particularly to swirled keratin debris [5], [6]. In particular, keratin debris is commonly encountered in SND specimen after chemoradiation. As it seems to be derived from tumor cells, it remains questionable whether this should be rated as a spectrum of viable tumor cells, notably if vital nuclei are present. To the best of our knowledge, only one recent publication investigated a large cohort comprising more than 140 cases of HNSCC for such keratin debris in LND after preceding chemoradiation [7]. Our data is in concordance with their results that evidence of keratin debris, even including vital nuclei, is not associated with a significantly worse prognosis. Thus, our data corroborates their finding and the statement that keratin debris should not be rated as viable tumor cells. One possible explanation is that, despite assumed origin from tumor cells, keratin debris probably is matured and loses its malignant biological capabilities. Scherpelz et al. also did report a significant worse prognosis when viable tumor cells were present in SND specimen [7]. This is in complete agreement with our results, defining viable tumor cells as highly atypical squamous cells with vital irregular nuclei, similar to untreated primary tumors or metastases. Of further interest was that the amount of viable tumor cells also appeared to have prognostic value. In our cohort we found that the larger the area of viable tumor cells, the worse the prognosis for locoregional recurrence rate. A possible explanation for this would be that a larger viable tumor area correlates with a greater tumor burden/worse response to treatment and thus a poorer prognosis. Interestingly, besides viable tumor cells extranodal extension, foamy histiocytes, and fibrosis did show an association with worse prognosis in an univariable analysis; however, multivariable analysis revealed only viable tumor cells as an independent poor prognostic factor. This suggests that the histopathological parameters shown to be significant predictors for a worse outcome in the univariable survival analysis, with the exception of viable tumor cells, seem to be influenced by confounders or showing significance by chance. In our subgroup analysis of oropharyngeal SCC, we showed a lower likelihood of viable tumors cells present in SND specimen with p16-positive tumors, which was associated with better survival. This is consistent with the previous work of our group [8].

Limitations of our study are its retrospective nature and the mix of HNSCC cases from different sites for proper statistical analysis. In contrast to Scherpelz and colleagues, our cohort did not include oral cavity SCC, as we included salvage cases only, i.e., cases after (chemo)radiation, whereas oral cavity SCC are usually operated on upfront. In conclusion, we can state that swirled keratin debris or pyknotic cells within necrosis should not be treated as an equivalent to viable tumor cells in pathological reports. Furthermore, none of the numerous investigated histopathological features occurring in the context of chemoradiation seem to be of prognostic value, except for depicting a reaction pattern to the therapy. However, we can corroborate viable tumor cells in lymph nodes after chemoradiation as an important prognostic factor. In addition, the area could be used as a surrogate for the tumor burden and thus further sub-stratify patients at even higher risk for a worse clinical course.

### Supplementary Information

Below is the link to the electronic supplementary material.

Supplementary material 1 (DOCX 16.0 kb)

Supplementary material 2 (TIFF 19767.6 kb)

## Data Availability

The datasets generated for this study can be obtained upon reasonable request by email to the corresponding author.
