# Supplementary material for: Histopathological Analysis of Nodal Disease After Chemoradiation Reveals Viable Tumor Cells as the most Important Prognostic Factor in Head and Neck Squamous Cell Carcinoma
Source: Head Neck Pathol. 2023 May 17;17(3):599–606. doi: 10.1007/s12105-023-01557-7 (PMC10514022; doi:10.1007/s12105-023-01557-7)
Supplement: Supplementary file 1 — Supplementary material 1 (DOCX 16.0 kb) [file 12105_2023_1557_MOESM1_ESM.docx]

**Table 2**

| Log Rank (Mantel Cox) for Variables | Sig (P<0.05*) |
| --- | --- |
| Viable Tumor cells |  |
| Locoregional recurrence-free survival | *3.53x10^-7^** |
| Distant metastasis-free survival | 0.00005* |
| Disease-specific survival | 0.00001* |
| Overall survival | 0.0001* |
| Viable tumor cells area  Locoregional recurrence-free survival  Distant metastasis-free survival  Disease-specific survival  Overall survival | *1.42x10^-7^**  0.00006*  0.0001*  0.0001* |
| Extranodal Extension |  |
| Locoregional recurrence-free survival | 0.004* |
| Distant metastasis-free survival | 0.210 |
| Disease-specific survival | 0.042* |
| Overall survival | 0.113 |
| Necrosis |  |
| Locoregional recurrence-free survival | 0,375 |
| Distant metastasis-free survival | 0,199 |
| Disease-specific survival | 0,975 |
| Overall survival | 0,457 |
| Necrosis area |  |
| Locoregional recurrence-free survival | 0,457 |
| Distant metastasis-free survival | 0,559 |
| Disease-specific survival | 0,404 |
| Overall survival | 0,588 |
| Swirled keratin debris |  |
| Locoregional recurrence-free survival | 0,785 |
| Distant metastasis-free survival | 0,563 |
| Disease-specific survival | 0,942 |
| Overall survival | 0,966 |
| Swirled keratin debris area (semi-quantitative) |  |
| Locoregional recurrence-free survival | 0,607 |
| Distant metastasis-free survival | 0,740 |
| Disease-specific survival | 0,861 |
| Overall survival | 0,614 |
| Foamy Histocytes |  |
| Locoregional recurrence-free survival | 0,031* |
| Distant metastasis-free survival | 0,030* |
| Disease-specific survival | 0,241 |
| Overall survival | 0,016* |
| Bleeding residues |  |
| Locoregional recurrence-free survival | 0,111 |
| Distant metastasis-free survival | 0,710 |
| Disease-specific survival | 0,263 |
| Overall survival | 0,456 |
| Fibrosis |  |
| Locoregional recurrence-free survival | 0,454 |
| Distant metastasis-free survival | 0,151 |
| Disease-specific survival | 0,147 |
| Overall survival | 0,038* |
| Fibrosis area (semi-quantitative) |  |
| Locoregional recurrence-free survival | 0,905 |
| Distant metastasis-free survival | 0,497 |
| Disease-specific survival | 0,450 |
| Overall survival | 0,136 |
| Elastosis |  |
| Locoregional recurrence-free survival | 0,145 |
| Distant metastasis-free survival | 0,879 |
| Disease-specific survival | 0,320 |
| Overall survival | 0,404 |
| Pyknotic cells |  |
| Locoregional recurrence-free survival | 0,396 |
| Distant metastasis-free survival | 0,844 |
| Disease-specific survival | 0,386 |
| Overall survival | 0,843 |
| Calcification |  |
| Locoregional recurrence-free survival | 0,111 |
| Distant metastasis-free survival | 0,103 |
| Disease-specific survival | 0,128 |
| Overall survival | 0,534 |
| Cholesterol crystals |  |
| Locoregional recurrence-free survival | 0,079 |
| Distant metastasis-free survival | 0,102 |
| Disease-specific survival | 0,075 |
| Overall survival | 0,105 |
| Multinucleal giant cells |  |
| Locoregional recurrence-free survival | 0,227 |
| Distant metastasis-free survival | 0,079 |
| Disease-specific survival | 0,248 |
| Overall survival | 0,154 |
|  |  |
| Note: all variables are binary (Y/N), except for the viable tumor cell area, necrosis area and fibrosis area, for which semi-quantitative variable were used. | |
