# Supplementary figures and images for: Histopathological Analysis of Nodal Disease After Chemoradiation Reveals Viable Tumor Cells as the most Important Prognostic Factor in Head and Neck Squamous Cell Carcinoma
Source: Head Neck Pathol. 2023 May 17;17(3):599–606. doi: 10.1007/s12105-023-01557-7 (PMC10514022; doi:10.1007/s12105-023-01557-7)

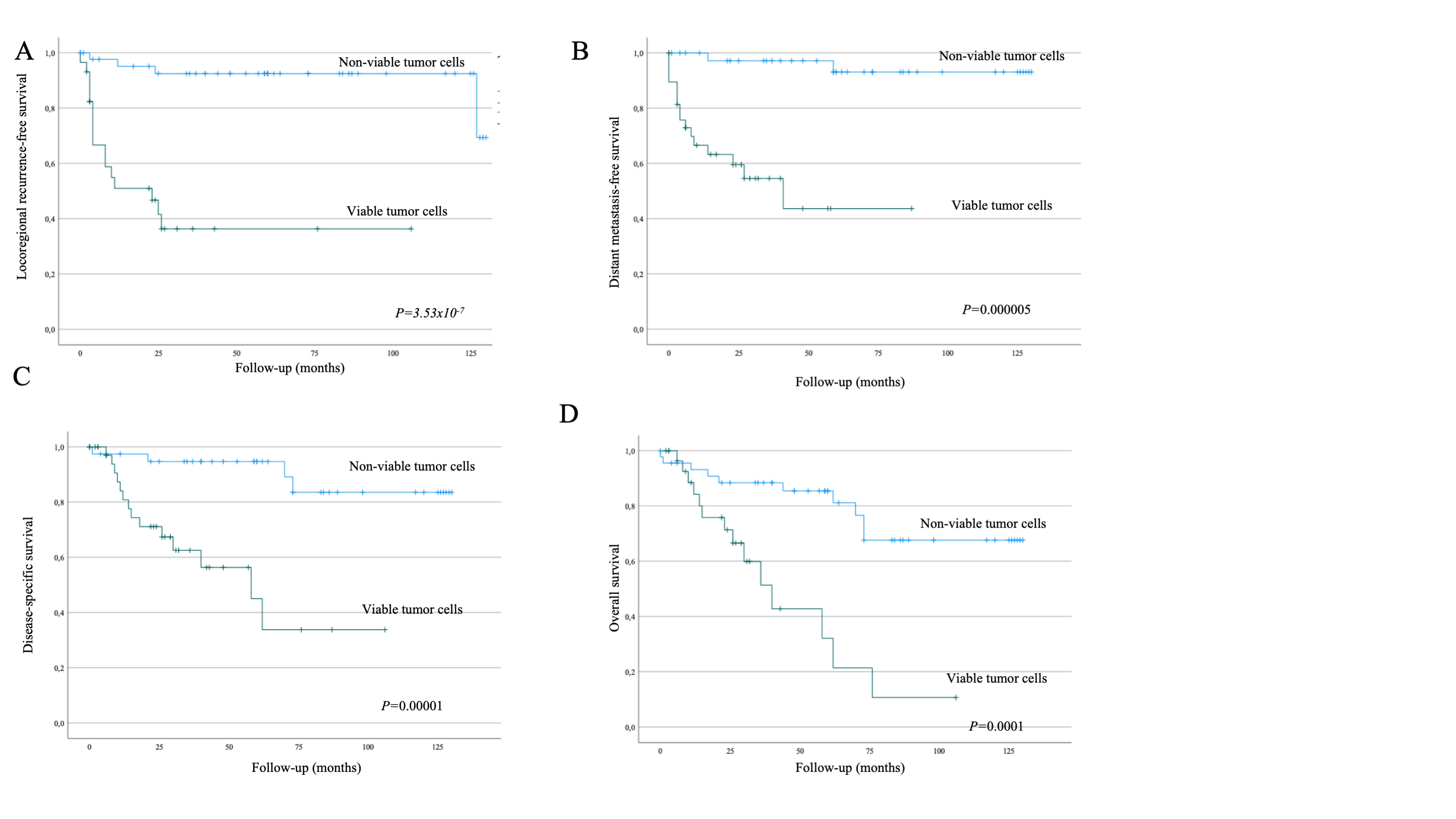

Supplement: Supplementary file 2 — Supplementary material 2 (TIFF 19767.6 kb) [file 12105_2023_1557_MOESM2_ESM.tiff]
